# Assessment of Habitat Suitability for the Invasive Vine *Sicyos angulatus* Under Current and Future Climate Change Scenarios

**DOI:** 10.3390/plants14172745

**Published:** 2025-09-02

**Authors:** Cui Xiao, Ji Ye, Haibo Zhang, Yonghui Qin, Ruihuan Yan, Guanghao Xu, Haili Zhou

**Affiliations:** 1 Satellite Environmental Application Center, Ministry of Ecology and Environment, Beijing 100093, China; 13811964453@163.com; 2 Institute of Applied Ecology, Chinese Academy of Sciences, Shenyang 110164, China; yanruihuan23@mails.ucas.ac.cn; 3 National Engineering Research Center of Eco-Environment in the Yangtze River Economic Belt, Beijing 100038, China; zhang_haibo@ctg.com.cn; 4 Hubei Key Laboratory of Rare Resource Plants in Three Gorges Reservoir Area, Yichang 443100, China; 5 Liaoning Agricultural Development Service Center, Shenyang 110001, China; 13909823173@139.com; 6 College of Forestry, Shenyang Agricultural University, Shenyang 110866, China; 2023240946@stu.syau.edu.cn

**Keywords:** *Sicyos angulatus*, invasive alien species, habitat suitability, maximum entropy (*MaxEnt*) model, geographic information system (ArcGIS), climate change

## Abstract

*Sicyos angulatus* L. is a rapidly spreading invasive alien vine that threatens natural and agricultural ecosystems globally. We collected occurrence data from 4886 sites and applied the maximum entropy (*MaxEnt*) model to assess current and future habitat suitability for *S. angulatus*. Future climate conditions were represented by low and high greenhouse gas concentrations under representative concentration pathways (i.e., RCP2.6 and RCP8.5, respectively). The *MaxEnt* model accurately predicted the distribution of *S. angulatus*, and the area under the receiver operating characteristic curve in the receiver operating characteristic test reached 0.921. Among the 19 climatic variables investigated, the best predictors for the distribution of *S. angulatus* were the precipitation in the driest month (with a contribution of 37.4%), annual precipitation (26.8%), average annual temperature (18.1%), and temperature seasonality (14.9%). Currently, the most suitable areas cover the central and eastern United States, parts of southern Europe, most Japanese islands, the majority of the Korean Peninsula, and eastern China, with a total area of 180.3 × 10^4^ km^2^ (1.2% of the Earth’s land area). During the 2050s and 2090s under RCP2.6 and RCP8.5, the most suitable regions worldwide are projected to expand by factors of 1.0 and 2.2, respectively. In particular, suitable areas might expand to higher-latitude regions and encompass previously unsuitable areas, such as Liaoning Province in Northeast China. These findings may aid in the surveillance and management of *S. angulatus*’ invasion globally.

## 1. Introduction

Burcucumber (*Sicyos angulatus* L.) is an annual, large climbing herbaceous vine of the Cucurbitaceae family, which originated in the United States [1,2]. With international economic integration over past decades, global trade and convenient transport among human society have promoted the expansion of *S. angulatus*. It was introduced as an ornamental garden plant to Europe during the 19th century [1]. Currently, *S. angulatus* has spread widely in most regions of Japan [3] and the Korean Peninsula [4,5], and has invaded small areas in China [6], Turkey [7,8], and certain European countries [9].

With highly rewarded nectary production, *S. angulatus* recruits various native pollinators to facilitate its seed dispersal and reproductive success during its invasion [2]. Its seeds are spread through wind, water, and animal and human transport, germinating throughout the growing season [10,11]. It develops rootstock with storage substances that support survival in nutrient-deficit or other harsh conditions [1,12]. Thus, it is highly adaptable to distinct habitats and exhibits high tolerance to varying soil properties, such as nutrient status and texture [10,12,13]. *S. angulatus* uses tendrils to acquire photosynthate and molecules (including proteins) from the phloem of its support and, subsequently, to strangle it to death [14].

*S. angulatus* can rapidly invade various niches of indigenous flora (such as *Salix alba* and *Populus nigra*) in the forests [15]. Owing to its rapid growth, *S. angulatus* outcompetes its neighbors for resources, with no weeds surviving nearby [1]. A single *S. angulatus* plant can threaten a 333 m^2^ cornfield, reducing yield by 50–80% [11]. Because of its notable threat to natural biodiversity and agricultural production, *S. angulatus* is considered a dangerous invasive alien species with a high risk-intensity rating [3,7].

*S. angulatus*’ great threats result not only from its high adaptability potential but also from climate change. Its seeds germinate at temperatures between 5 and 40 °C, with an optimum growth temperature of 24–25 °C. In addition, it prefers high moisture [16,17,18] and thrives in riparian or frequently flooded humid areas [8]. Global change has been ongoing for decades, and alterations in precipitation patterns and warming effects are expected to increase substantially in the future. The invasive range of *S. angulatus* may expand with increasing temperatures and precipitation levels [19]. Analyzing the potential distribution of *S. angulatus* under current and future climate change scenarios constitutes the basis for risk assessment and management decisions. These measures must be taken before or at the early stage of *S. angulatus*’ invasion at the local, regional, and global scales [20,21].

Ecological niche models have been adopted to better understand the relationships between invasive aliens and bioclimatic conditions [22,23]. Based on the actual distribution of alien species and available environmental datasets, various algorithms can determine the ecological requirements and survival ranges of aliens globally [24,25]. Such habitat suitability models include logistic regression (LR), the Mahalanobis distance (MD), the genetic algorithm for rule set production (GARP), and maximum entropy (*MaxEnt*) [24,25,26,27,28]. Among these habitat suitability models, the *MaxEnt* model was created to predict potential geographic niches by deriving the optimal distribution of maximum entropy values (the most spread out or closest to a uniform pattern). The model is subject to constraints from presence-only datasets of georeferenced occurrence locations and corresponding climatic variables [29]. In particular, the *MaxEnt* model offers advantages in providing quantitative descriptions of habitat simulations and requirements via the representative concentration pathway (RCP) of the corresponding climate change scenario [6,20,24,30,31]. For instance, the *MaxEnt* model has been applied to predict that the suitable areas of invasive weeds (such as *Ageratina adenophora*) may expand globally, particularly in regions with high elevation (3000–3500 m) [20,32]. Further, the *MaxEnt* model reveals that temperature and precipitation are important climatic variables influencing the invasive success of *Bidens pilosa* (a globally invasive weed) [33].

In this study, we applied the *MaxEnt* model to analyze the potential distributions of *S. angulatus* in relation to key climatic conditions. Our objectives were to clarify the following: (1) What are the climatic thresholds associated with the dominant climatic variables influencing the distribution of *S. angulatus* at the local, regional, and global scales? (2) Which regions are most susceptible to the expansion of *S. angulatus* under varying RCPs? Our findings provided both empirical and theoretical foundations for assessing the invasion risk of *S. angulatus* under two future climate change scenarios, namely RCP2.6 and RCP8.5, which correspond to low and high estimates of potential greenhouse gas emissions, respectively. Our results could inform strategies to protect vulnerable regions and manage the global invasion risk of *S. angulatus*.

## 2. Results

### 2.1. Assessment of Prediction Accuracy via the MaxEnt Model

**Figure 1:**
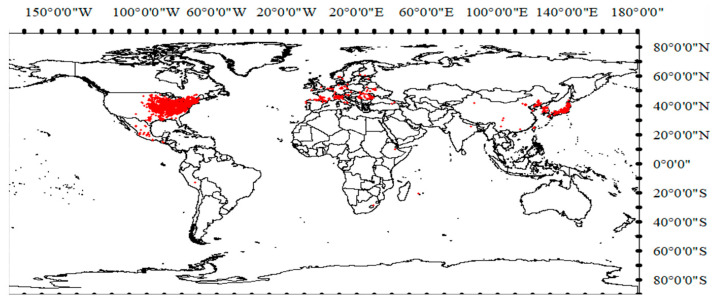
Locations of occurrence records of *S. angulatus*, represented by red points.

In this study, a total of 4886 sites were selected that formed the basis for our species distribution modeling (Figure 1).

**Figure 2:**
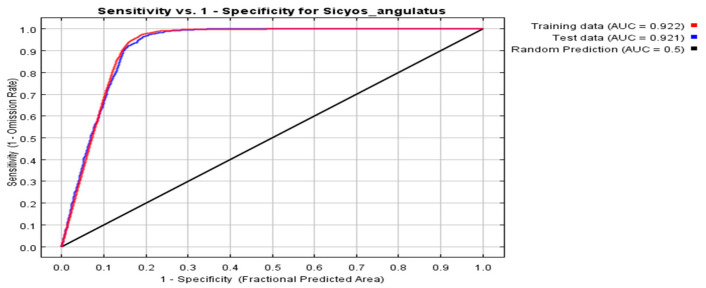
ROC curves of sensitivity vs. specificity for *S. angulatus*.

The performance of the *MaxEnt* model in predicting potential habitat suitability for *S. angulatus* was assessed via the area under the receiver operating characteristic curve (AUC) through the receiver operating characteristic (ROC) test. The AUC values derived by the *MaxEnt* model for the training and test datasets were 0.922 and 0.921, respectively (Figure 2).

AUC values exceeding 0.9 indicate an outstanding or highly accurate test [34]. These results suggest that our *MaxEnt* model is highly accurate for identifying potentially suitable areas for *S. angulatus* globally. To assess the variability in the results, we incorporated 10 replicates in the modeling process and calculated confidence intervals to determine the reliability of our habitat suitability assessments (Figure S1). By acknowledging uncertainties, we aimed to provide a more transparent and comprehensive analysis of the potential distribution of *S. angulatus*.

### 2.2. Key Climatic Variables Influencing the Distribution of S. angulatus

A total of 19 climatic variables (Bio1–Bio19) were chosen in this study (Table 1). As revealed by the jackknife test, climatic determinants notably contributed to the potential suitability of *S. angulatus* habitats worldwide.

**Figure 3:**
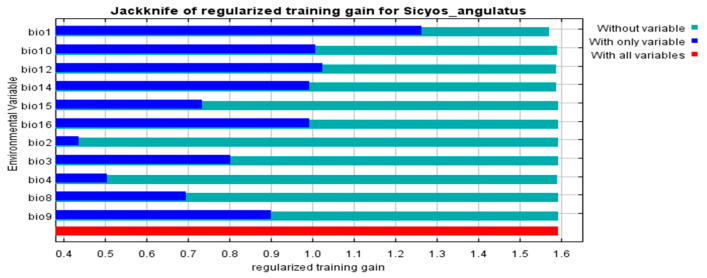
Analysis of variable importance via the jackknife test based on a regularized training gain. Bio1, average annual temperature (°C); Bio2, mean diurnal range (mean monthly value of (maximum temperature−minimum temperature)) (°C); Bio3, isothermality (BIO2/BIO7) (× 100); Bio4, temperature seasonality (standard deviation × 100); Bio5, maximum temperature in the warmest month (°C); Bio6, minimum temperature in the coldest month (°C); Bio7, temperature annual range (BIO5–BIO6) (°C); Bio8, mean temperature in the wettest quarter (i.e., a period of three months) (°C); Bio9, mean temperature in the driest quarter (°C); Bio10, mean temperature in the warmest quarter (°C); Bio11, mean temperature in the coldest quarter (°C); Bio12, annual precipitation (mm); Bio13, precipitation in the wettest month (mm); Bio14, precipitation in the driest month (mm); Bio15, precipitation seasonality (coefficient of variation); Bio16, precipitation in the wettest quarter (mm); Bio17, precipitation in the driest quarter (mm); Bio18, precipitation in the warmest quarter (mm); and Bio19, precipitation in the coldest quarter (mm).

The four most important climatic variables accounted for 97.2% of the cumulative contributions to the distribution of *S. angulatus*. These variables were Bio14 (precipitation in the driest month) with a contribution of 37.4%, Bio12 (annual precipitation; 26.8%), Bio1 (average annual temperature; 18.1%), and Bio4 (temperature seasonality, 14.9%). Therefore, the distribution of *S. angulatus* is influenced more notably by precipitation (with a combined contribution of 64.2% by Bio14 and Bio12) than by temperature (with a combined contribution of 33.0% by Bio1 and Bio4). Using the jackknife test to evaluate the permutation importance of each variable, we found that Bio1 (average annual temperature) was the most important determinant of the potential distribution of *S. angulatus*, followed by Bio12 (annual precipitation), Bio10 (mean temperature in the warmest quarter), Bio14 (precipitation in the driest month), and Bio16 (precipitation in the wettest quarter). The variable with the lowest weight was Bio2 (mean diurnal range) (Figure 3).

**Figure 4:**
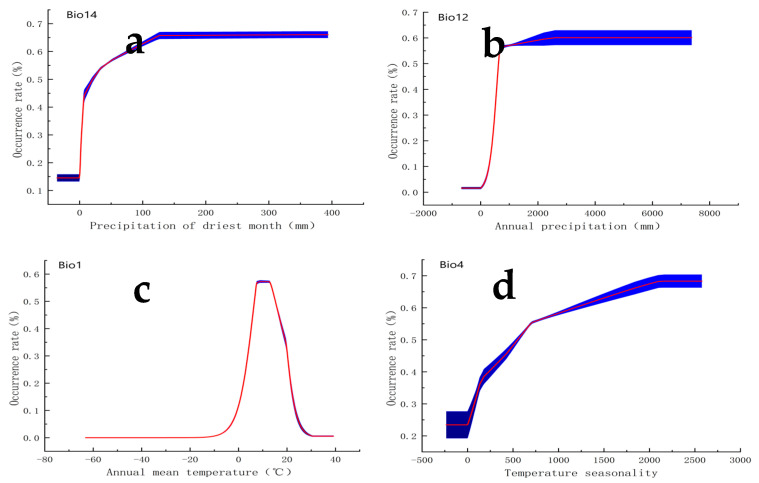
Response curves of the key climatic variables adopted in the *MaxEnt* model for the distribution of *S. angulatus* habitats. (**a**) Bio1, average annual temperature (°C); (**b**) Bio4, temperature seasonality (standard deviation × 100); (**c**) Bio12, annual precipitation (mm); (**d**) Bio14, precipitation in the driest month (mm). The red and blue lines represent means and standard deviations (*n* = 10), respectively.

We generated curves of the best-fit habitat in response to the four important variables that contributed the most to the *MaxEnt* model. The correlations demonstrated that the suitable ranges of the climatic variables for *S. angulatus* (existence probability exceeding 0.5) are as follows: >20.9 mm for Bio14 (precipitation in the driest month), >632.7 mm for Bio12 (annual precipitation), 6.6–15.0 °C for Bio1 (average annual temperature), and >552.7 for Bio4 (temperature seasonality, standard deviation × 100) (Figure 4).

### 2.3. Current Habitat Suitability for S. angulatus According to the MaxEnt Model

**Figure 5:**
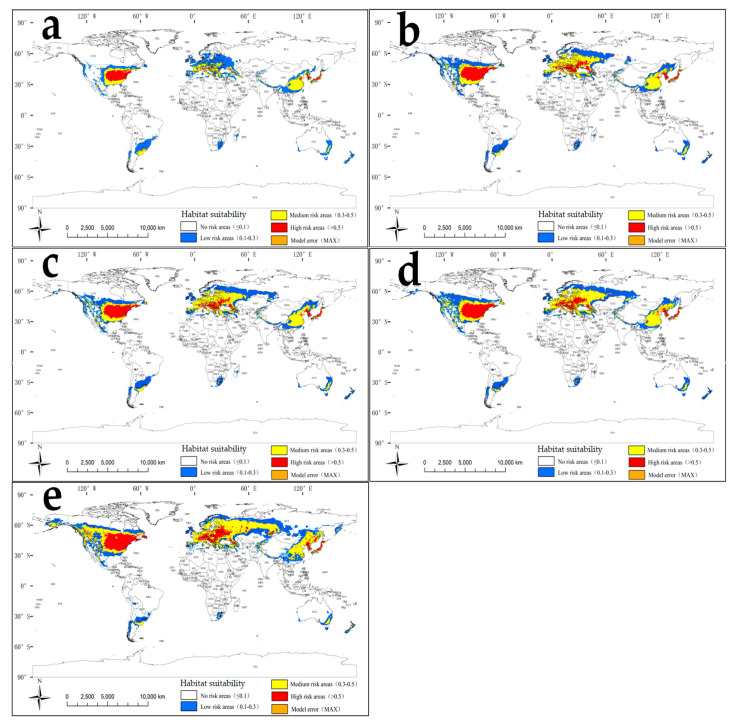
Habitat suitability for *S. angulatus* under current and future climate change scenarios based on different RCPs. (**a**) Contemporary era (1970–2000); (**b**) 2050s under RCP2.6; (**c**) 2090s under RCP2.6; (**d**) 2050s under RCP8.5; (**e**) 2090s under RCP8.5.

Globally, during the contemporary era (1970–2000), highly suitable habitat for *S. angulatus* (Figure 5a) was concentrated in the central and eastern United States, southern and eastern Europe (extending into western Russia), and East Asia (the Korean Peninsula, Japanese islands, and eastern China). In particular, the core areas of high suitability mainly covered the southeastern parts of the Midwest region and northern parts of the Southeast region in the United States. Moderate habitat suitability for *S. angulatus* mainly occurred in the southern parts of the Southeast region and the central and eastern parts of the Midwest region of the United States, southeastern China, southern Europe, and southeastern South America (e.g., Argentina and Uruguay).

Highly suitable habitat occupied a total area of 180.3 × 10^4^ km^2^, accounting for 1.2% of the Earth’s land surface (Table 2). Moderately suitable habitat encompassed a total area of 436.6 × 10^4^ km^2^, accounting for 2.9% of the Earth’s land surface. Most remaining surface areas were affiliated with poorly suitably or unsuitable habitats for *S. angulatus*.

**Figure 6:**
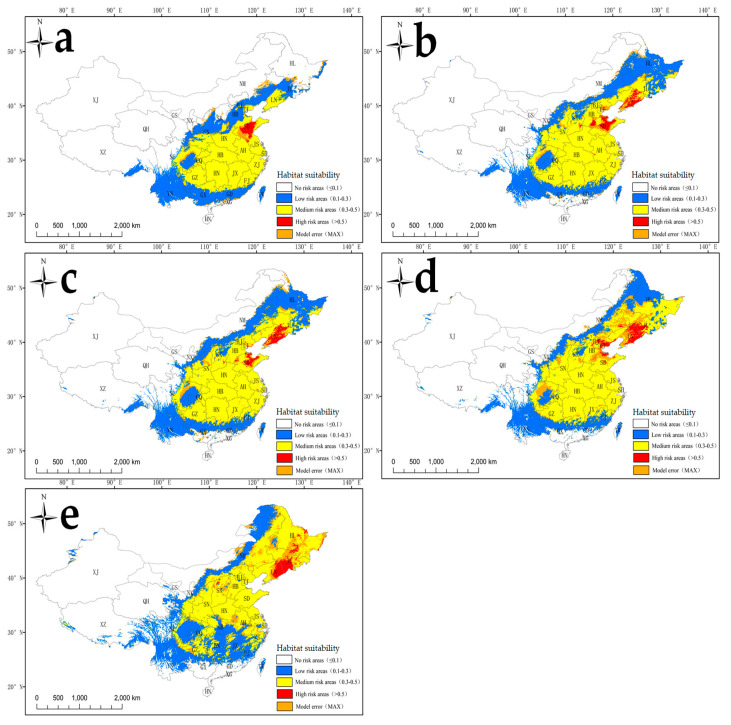
Habitat suitability for *S. angulatus* in mainland China under current and future climate change scenarios based on different RCPs. (**a**) Contemporary era (1970–2000); (**b**) 2050s under RCP2.6; (**c**) 2090s under RCP2.6; (**d**) 2050s under RCP8.5; (**e**) 2090s under RCP8.5.

In particular, mainland China encompassed a large area of high suitability in central Shandong Province, northwestern Jiangshu Province, and southwestern and northeastern Anhui Province, which occupied a total area of 6.0 × 10^4^ km^2^ and accounted for 0.6% of the surface area of mainland China (Figure 6a, Table 2). In addition, moderately suitable habitat occupied an area of 173.4 × 10^4^ km^2^ and accounted for 18.1% of the surface area of the Chinese mainland. Such habitats were distributed mainly in central, eastern, and northeastern China, including Liaoning, Shandong, Jiangsu, Shanghai, Zhejiang, Fujian, Anhui, Jiangxi, Henan, Hubei, and Hunan Provinces.

**Figure 7:**
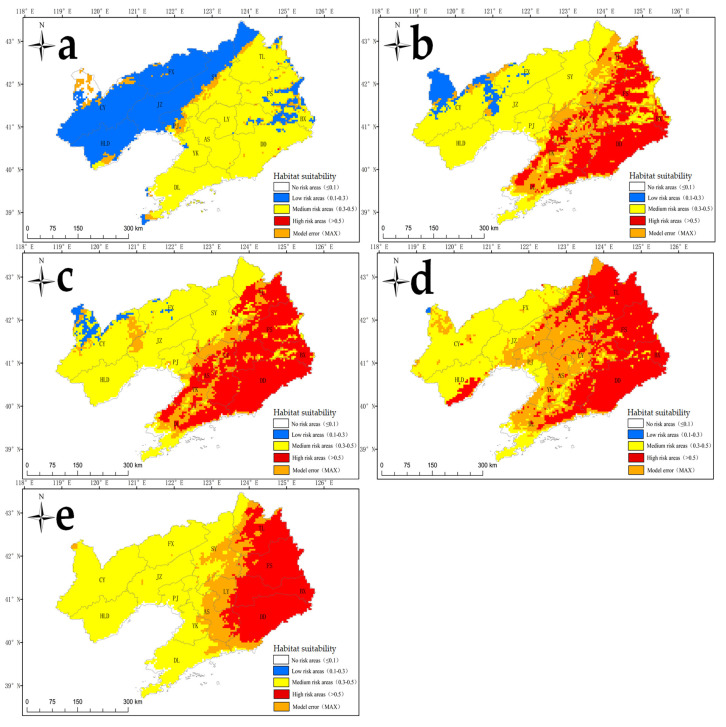
Habitat suitability for *S. angulatus* in Liaoning Province of Northeast China under current and future climate change scenarios based on different RCPs. (**a**) Contemporary era (1970–2000); (**b**) 2050s under RCP2.6; (**c**) 2090s under RCP2.6; (**d**) 2050s under RCP8.5; (**e**) 2090s under RCP8.5.

Additionally, Liaoning Province contained no highly suitable areas for *S. angulatus*, and its moderately suitable habitat was predicted to cover 7.8 × 10^4^ km^2^, accounting for 52.2% of the total terrestrial area, including the cities of Tieling, Shenyang, Fushun, Liaoyang, Benxi, Dandong, Anshan, Yingkou, and Dalian (Figure 7a, Table 2).

### 2.4. Future Habitat Suitability for S. angulatus According to the MaxEnt Model

To better understand the future potential spread of *S. angulatus* on a worldwide scale, we applied the *MaxEnt* model to simulate the suitable habitat for *S. angulatus* during the 2050s and 2090s based on the predicted climate dataset from the future climate change scenarios (i.e., RCP2.6 and RCP8.5, respectively) (Figure 5b–e; Table 2). During the 2050s (RCP2.6), highly and moderately suitable areas worldwide were predicted to increase by 100.8% and 75.1%, respectively (Figure 5b, Table 2). In the United States during the 2050s (RCP2.6), for instance, highly suitable regions may expand and cover most areas of the northwestern region and become sparsely distributed in the southwestern region (Figure 5b). In Europe during the 2050s (RCP2.6), highly suitable regions may unprecedentedly occur in Germany, Austria, Romania, Ukraine, Moldova, Bulgaria, and Belarus (Figure 5b). During the 2090s (RCP8.5), the risk of highly suitable invasion may further expand to unprecedented areas, including southwest Canada, south Russia, and northeast China (Figure 5e). In particular, moderate suitability regions may even cover the southern parts of Russia’s far east during the 2090s (RCP8.5) (Figure 5e). These increases in highly and moderately suitable areas were projected to occur worldwide, with 2.2- and 2.0-fold increases, respectively (Figure 5e, Table 2).

Lower invasion intensity was predicted in China than at the global scale under future climate change conditions (Figure 6b–e, Table 2). For instance, from the 2050s to the 2090s, the areas of highly suitable habitat in mainland China may increase by 0.7–0.9-fold under RCP2.6 and by 1.3–1.5-fold under RCP8.5 compared with those in the contemporary era (1970–2000). Specifically, from the 2050s to the 2090s, the areas of highly suitable habitat in Liaoning Province may account for 27.3–34.2% of the total area under RCP2.6 and 27.0–38.8% of the total area under RCP8.5, whereas no occurrence was found in the contemporary era (1970–2000) (Figure 7b–e, Table 2). Furthermore, even under the low-level climate change scenario (RCP2.6), *S. angulatus* may cover the entire area of Liaoning Province (Figure 7b,c).

## 3. Discussion

*S. angulatus* is highly adaptable to various environments, posing great threats to natural and agricultural ecosystems worldwide [1,2,3,4,7]. By applying the *MaxEnt* model, combined with its occurrence data as well as climatic variables, we predicted the current and future habitat suitability for *S. angulatus* at the global, regional, and local scales. In the absence of effective interference, *S. angulatus*’ invasion may expand notably to relatively high latitudes or the inner mainland, reaching previously unsuitable niches in the future.

### 3.1. Performance of the MaxEnt Model

The *MaxEnt* model is widely employed to determine the potential distribution of alien species by calculating the maximum entropy of a stable system, which consists of its occurrence locations and corresponding climatic conditions [20,21]. The high AUC values for the training and test datasets (Figure 3) supported the accuracy and stability of the established ArcGIS-based *MaxEnt* model as an effective approach to simulate the suitable niche map for *S. angulatus*.

Despite the high accuracy of the *MaxEnt* model, uncertainties in the projections were observed, especially for Bio4 (temperature seasonality), Bio12 (annual precipitation), and Bio14 (precipitation in the driest month) (Figure 4). These uncertainties arise from variability in the input data, model parameters, and the inherent stochasticity in the modeling process. To address these uncertainties, we employed several strategies. A total of 4886 sites of *S. angulatus* from the Global Biodiversity Information Facility (GBIF) and the National Specimen Information Infrastructure (NSII), and our surveys were screened to ensure that only one distribution data point existed within a 5 km radius. Multicollinearity between variables can lead to overfitting of the species distribution model, resulting in a model that is too constrained and unreliable. Therefore, Pearson’s correlation was used to avoid multicollinearity among the 19 climatic variables [22,24,25,26]. By conducting 10 iterations of the model, we assessed the variability and calculated confidence intervals, which could aid in understanding the range of possible outcomes. We fine-tuned the *MaxEnt* model by experimenting with various regularization multiplier (RM) values and feature classes (FCs) [35]. Specifically, we considered RM values ranging from 0.5 to 4 at 0.5 intervals combined with different FCs (e.g., Linear, Quadratic, Hinge, Product, and Threshold), allowing us to identify the most robust model parameters. We applied an RM equal to 0.5 and an FC as the LQHPT for the final model, which had the lowest Delta AICc value of 0. Additionally, the *MaxEnt* model did not consider non-climatic variables, which influenced the accuracy of prediction [32]. Therefore, it would be useful to improve the model’s accuracy by integrating additional variables associated with soil traits, plant communities, and land use.

### 3.2. Importance of Precipitation for S. angulatus’ Habitat Suitability

Precipitation is assumed to significantly influence the invasive success of non-native species [36,37]. *S. angulatus* tends to emerge under humid conditions and is highly sensitive to water deficit [8,13]: no seedlings survive extreme water deficiency (12.5% moisture availability based on volumetric pot water content) and the species fails to tolerate salinity levels of 6 and 12 dSm^−1^. These traits suggest that its expansion should be constrained in arid climates, whereas high precipitation can increase soil moisture and thereby alleviate osmotic and salinity stress. In this study, the jackknife analysis demonstrated that its distribution is notably influenced by precipitation.

Specifically, Bio14 (precipitation in the driest month) and Bio12 (annual precipitation) were the most important climatic variables in determining the potentially suitable habitats of *S. angulatus*. We estimated their minimum thresholds for the growth of *S. angulatus*, which exceeded 20.9 mm for Bio14 and 632.7 mm for Bio12. Since the 1970s, the central and eastern United States have received homogeneous and large volumes of precipitation and thus are becoming wetter [38,39]. In these regions, dry-season precipitation typically ranges from 2.6 to 3.1 mm per day—well above the Bio14 threshold—and exceeds that in the remaining parts of the country [40]. Additionally, these areas receive ~800 mm of annual precipitation [41], which is greater than the Bio12 threshold. For the Korean Peninsula and Japan, the annual precipitation amount is usually greater than 1000 mm [42,43,44], creating favorable moisture for *S. angulatus*’ growth. In addition, most regions of Japan experience three rainy seasons separated by short-term droughts, and the average drought occurrence is 0.89 events per observation per year [45]. As revealed by the *MaxEnt* model, highly suitable habitat was mainly concentrated in the abovementioned regions.

### 3.3. Importance of Temperature for S. angulatus’ Habitat Suitability

Temperature is a key determinant of the distribution of alien species [37,46]. *S. angulatus* requires adequate heat accumulation for growth, with an optimum growth temperature of 24–25 °C [8]. The *MaxEnt* model revealed that the most important temperature variable influencing *S. angulatus* is Bio1 (average annual temperature), with a suitable range of 6.6–15.0 °C. In the central and eastern United States, the summer mean surface air temperature mostly ranges from 18 to 24 °C [47], and the average annual temperature can reach 12.7 °C [41]. Korea and Japan exhibit summer mean surface air temperatures of 18–25 °C [48] and mean annual temperatures of 5.9–15.4 °C [42,44]. Thus, these regions provide warm growing-season conditions near *S. angulatus*’ physiological optimum while falling within the modeled Bio1 range.

The abovementioned suitable temperature range indicates that *S. angulatus* does not thrive in climate zones that are extremely cold or hot. Such intermediate climates typically exhibit pronounced temperature variability with distinct and well-defined seasons [49]. The regions where *S. angulatus* naturally occurs or where it has severely invaded typically demonstrate widely varying minimum (winter) and maximum (summer) temperatures throughout the year. Accordingly, Bio4 (temperature seasonality) strongly contributed to habitat suitability for *S. angulatus*, with an optimal value of > 552.7 (standard deviation × 100). Prior studies show that temperature seasonality shapes climatic conditions related to the physiological performance, phenotypic plasticity, and fitness-related traits of alien species [6,49,50].

### 3.4. Invasion Risk and Management Under Current and Future Climate Scenarios

While the current distribution maps of invasive species may capture the most favorable conditions, ongoing climate change (warming and wetting) can alter the distributions of habitat suitability [33]. Such climate changes may facilitate further expansion of *S. angulatus*, disrupting natural and agricultural ecosystems and imposing substantial socioeconomic costs [20,27]. It is reported that a 10% infestation of cornfields in highly suitable habitats can result in economic losses exceeding RMB 430 million in Liaoning Province, northeast China [10]. Consequently, concerns have been raised regarding the accelerated invasion of *S. angulatus* under climate change [5]. In particular, as shown by the *MaxEnt* model, *S. angulatus*’ invasion is highly constrained by precipitation levels during the dry season or at the annual scale, by the average annual temperature, and by temperature seasonality. As these climatic variables are increasingly affected by temperature and precipitation extremes [51,52,53,54], *S. angulatus* may occur at a high risk of unpredictable weather patterns.

Under future climatic conditions (Figure 5b–e; Table 2), highly suitable regions for *S. angulatus* are projected to increase by more than twofold during the 2050s–2090s. This expansion is noticeable in North America and Europe, as the regions at latitudes of approximately 40–60° N might experience increases in precipitation and temperature [55,56]. Such climate changes create conditions conducive to *S. angulatus*’ invasion and spread. The shift in suitability is more pronounced under the high carbon emission scenario (i.e., RCP8.5). As shown in Table 3, the areas of habitat suitability of *S. angulatus* vary across alternative continents and future climate scenarios. For instance, Asia experienced the greatest expansion amplitude from RCP2.6 to RCP8.5, increasing by 16.17% and 30.15% during the 2050s and 2090s, respectively. For mainland China, substantial expansion was projected by the 2050s–2090s (Figure 6b–e; Table 2), with highly suitable niche areas potentially expanding by 0.7–0.9- and 1.3–1.5-fold under RCP2.6 and RCP8.5, respectively.

Such projections align with the species’ traits, such as generalist pollination [2] and tolerance of varying soil properties [10,12,13]. Therefore, this species can capitalize on climate changes by outcompeting native flora for sunlight and nutrients [1] and rapidly occupying various niches, resulting in the extinction of nearby plants in both natural and agricultural ecosystems in the future [10,11].

To mitigate the invasion of *S. angulatus*, it is imperative to enhance management efforts such as risk-based monitoring and quarantine in high-suitability regions identified by the *MaxEnt* model. Early detection and rapid response are critical to the invasion of *S. angulatus*. Reducing propagule pressure (e.g., through sanitation of machinery and seed lots) and strengthening biosecurity at transport corridors can further limit the spread. Native plant communities offer an effective biotic resistance: Manipulating the community assembly (e.g., promoting diversity) and optimizing combination of seed density can suppress *S. angulatus* [57]. The interaction between native vegetation communities and soil nutrient status is particularly important. For example, *Lespedeza cuneata* combined with *Pennisetum alopecuroides* can efficiently acquire resources and reduce nutrient availability for *S. angulatus*. In contrast, *Lespedeza bicolor* combined with *Lactuca indica* performs well under nutrient deficiency, effectively limiting *S. angulatus*’ invasion in unfertilized soil [12]. These effects are linked to functional traits such as shoot and leaf-area allocation and root density. Diverse and functionally complementary native plantings can maintain high resource-use efficiency and adapt to various niches, making them more effective than simple communities in resisting the invasion success of *S. angulatus*. Therefore, establishing a dense, diverse, and invasion-resistant native plant community, alongside sustained surveillance and rapid response protocols, is a practical and effective strategy for controlling *S. angulatus* [57].

## 4. Materials and Methods

### 4.1. Data Acquisition

#### 4.1.1. Occurrence Records of *S. angulatus* Worldwide

Occurrence records of *S. angulatus* worldwide were obtained from the GBIF (Occurrence Download: https://doi.org/10.15468/dl.quduju (accessed 10 April 2024)) and the NSII (Occurrence Download: https://doi.org/10.15468/kmob80 (accessed 10 April 2024)). Additionally, from 2023 to 2024, we conducted a field investigation of *S. angulatus* across 14 cities in Liaoning Province (38°43′–43°26′ N, 118°53′–125°46′ E), China. During the survey, we recorded the presence of *S. angulatus* and the specific locations of each sample (Figure S2). The survey data were subsequently screened to remove duplicate records, and the longitude and latitude of each record were verified via Google Earth. Ultimately, a total of 4886 sites were retained globally, which formed the basis for our species distribution model (Figure 1). The proportions of the data from the GBIF, the NSII, and our own survey were 50.8%, 28.1%, and 21.1%, respectively.

The longitude and latitude of the occurrence records were determined via Google Earth. We used ArcGIS (version 10.2) software to visualize the *MaxEnt* model’s output and to derive the distance between the records and the center, thereby ensuring that one distribution point was closest to the center in each censored grid. To enhance the reliability of our projections, we carefully examined the spatial accuracy of each occurrence record. The spatial accuracy of the GBIF and the NSII data was assessed on the basis of the metadata provided with each record. Most occurrences included GPS coordinates with a precision range between 5 and 100 m. For the additional field surveys in Liaoning, professional experts on herbs conducted the investigation. We employed high-precision GPS devices (HeFei ZhuoLin Electronic Technology Co. Ltd., Hefei, China) to ensure spatial accuracy within 5 m. This rigorous approach allowed for the acquisition of more reliable and fine-scale spatial data, contributing to the robustness of the model.

Despite these efforts, we acknowledge certain limitations in our dataset. Records from the GBIF and the NSII, while extensive, vary in precision and completeness. Certain areas, especially less accessible regions, may be underrepresented due to sampling biases. To prevent spatial autocorrelation, sampling bias, and model overfitting, we employed ArcGIS to import the CSV file containing the data points and project these points onto the map. Then, we created a 5 km radius buffer zone for each point. Via the Explode Multipart Feature function from Advanced Editing, we separated the buffer zones into individual patches. And then, we joined the point file with the Buffer file. Next, we assigned corresponding values to points that occurred in patches that overlapped or intersected. Finally, we removed points with identical values via the Delete Identical Tool in Data Management. Ultimately, we ensured that only one distribution data point existed within a 5 km radius and retained 2083 points for further analyses.

#### 4.1.2. Climatic Variables

The data on the climatic variables were downloaded from the WorldClim database [58]. We chose 19 climatic variables (Bio1–Bio19) (Table 1), including contemporary variables for 1970–2000 and those predicted for 2040–2060 and 2080–2100 under both low and high carbon emissions (i.e., RCP2.6 and RCP8.5, respectively). These 19 variables were extracted from the corresponding layers via the ArcGIS 10.2 statistical package (Table 1). The climatic variables were accessed in raster format with a spatial resolution of 30 arc-minutes (~1 km^2^).

Pearson correlation’s analysis was conducted on the climate data of the 2083 points (Figure S3). Given that certain pairs of climate variables exhibited Pearson correlation coefficients (r) higher than 0.8, one of each pair was deleted. The highly correlated variable pairs included Bio10 (mean temperature in the warmest quarter) vs. Bio5 (maximum temperature in the warmest month) (r = 0.95), Bio1 (average annual temperature) vs. Bio6 (minimum temperature in the coldest month) (r = 0.85), Bio4 (temperature seasonality) vs. Bio7 (temperature annual range) (r = 0.94), Bio16 (precipitation in the wettest quarter) vs. Bio13 (precipitation in the wettest month) (r = 0.98), Bio1 (average annual temperature) vs. Bio11 (mean temperature in the coldest quarter) (r = 0.92), Bio14 (precipitation in the driest month) vs. Bio19 (precipitation in the coldest quarter) (r = 0.91), Bio16 (precipitation in the wettest quarter) vs. Bio18 (precipitation in the warmest quarter) (r = 0.89), and Bio14 (precipitation in the driest month) vs. Bio17 (precipitation in the driest quarter) (r = 0.99) (Figure S3). The choice of variables was justified on the basis of their ecological relevance to the distribution of *S. angulatus* according to Araújo et al. (2019) [59]. For instance, for the Bio10 vs. Bio5 pair, we removed Bio5 but retained Bio10 because the latter is more closely related to the accumulated temperature, which greatly impacts the distribution of *S. angulatus* [5,10]. Thereafter, only 11 environmental variables (i.e., Bio1, Bio2, Bio3, Bio4, Bio8, Bio9, Bio10, Bio12, Bio14, Bio15, and Bio16) were retained in our final model, and their importance was analyzed via the jackknife test based on a regularized training gain (Figure 3).

### 4.2. Methods

#### 4.2.1. Model Establishment and Accuracy Evaluation

The data for the distribution of *S. angulatus* across 2083 sites and 11 corresponding climatic variables were used to develop the *MaxEnt* model. The jackknife analysis was employed to evaluate the reliability of the model and the relative importance of each climatic variable in explaining the distribution of *S. angulatus* [6,24,31]. For model validation, 75% of the distribution points were randomly chosen for model training, and the remaining 25% were reserved as a test dataset (to evaluate the predictive power). We measured the prediction accuracy of the model on the basis of the area under the threshold-independent ROC curve, which was used to plot the omission error (*x*-axis) versus the sensitivity (*y*-axis). The AUC was selected to estimate the accuracy of the model and ranged from 0 to 1. An AUC value less than 0.50 suggests that the model performance is no better than random, whereas values closer to 1.00 indicate perfect model performance. AUC values greater than 0.9 correspond to an outstanding or perfect test (i.e., highly suitable habitat); 0.8–0.9, excellent; 0.7–0.8, acceptable; 0.6–0.7, poor; and lower than 0.5, a random prediction of presence and absence or an inaccurate test (i.e., absolutely unsuitable habitat) [34,60].

We incorporated 10 replicates in our modeling process to assess the variability in the results (Figure S1). We assessed uncertainty by calculating confidence intervals for the predicted probabilities to provide a clear picture of the reliability of our habitat suitability assessments. By implementing 500 iterations of the model with different random seeds, we could better understand the stability and reliability of our predictions. To assess the uncertainty associated with the model’s predictions, we conducted sensitivity analyses by varying key parameters within the *MaxEnt* model, such as regularization multipliers and the maximum number of background points. In this study, how different configurations influence the predictions was assessed, and the parameters influencing the model outcomes were optimized. We implemented several *MaxEnt* model configurations, including the use of the 10th percentile presence probability of the species, a 10-fold cross-validation method, 10 repeat runs, 10,000 background points, a complementary log-log (clog-log) output format, 500 iterations, the generation of response curves, and an examination of jackknife importance in the final optimized species distribution model.

#### 4.2.2. Classification of Habitat Suitability

ArcGIS 10.2 software was used to extract the predicted values of the *MaxEnt* model and transform these values into raster pixel values, which indicate the survival rates of *S. angulatus* in each raster grid (potential distribution map). The predicted probabilities of the presence of *S. angulatus* were classified as highly suitable habitats, with values of 0.5–1; moderately suitable habitats, with values of 0.3–0.5; poorly suitable habitats, with values of 0.1–0.3; and unsuitable habitats, with values of 0–0.1 [24].

#### 4.2.3. Habitat Suitability Expectations Under Future Climate Change

On the basis of the abovementioned classification of habitat suitability, we applied the *MaxEnt* model and ArcGIS 10.2 to predict the potential distribution of suitable habitats for *S. angulatus* during the 2050s (2041–2060) and 2090s (2081–2100) under different future climate scenarios at various scales, i.e., the global scale, the regional scale (China), and the local scale (Liaoning Province). Two RCP climate scenarios, i.e., RCP2.6 and RCP8.5, were used to denote low and high greenhouse gas emissions, respectively [24]. Future variable data were derived via the Climate Community Climate System Model version 4 (CCSM4) [20].

## 5. Conclusions

Using the *MaxEnt* model, we projected the current and future suitable habitats for *S. angulatus* globally. This invasive species prefers wet and warm conditions and demonstrates high invasion risks under climate change. It is essential to implement early detection efforts and decisive control measures in highly suitable areas, including southeastern China, Europe, and North America. *S. angulatus* can outcompete native species for resources and habitats, thereby altering the community structure and potentially leading to the extinction of nearby plants. Future research should integrate socioeconomic impact assessments and predictive modeling under combined climate and land use scenarios to better understand and mitigate these impacts.

## Figures and Tables

**Table 1:** Analysis of variable importance was performed via the jackknife test, based on a regularized training gain.

Labels	Climatic Variables	Percent Contribution (%)	Permutation Importance (%)
Bio1	Average annual temperature (°C)	18.1 ± 0.7	57.5 ± 2.3
Bio2	Mean diurnal range (mean monthly value of (maximum temperature−minimum temperature)) (°C)	0.2 ± 0.2	0.4 ± 0.1
Bio3	Isothermality (BIO2/BIO7) (×100)	1.4 ± 0.7	18.7 ± 3.2
Bio4	Temperature seasonality (standard deviation ×100)	14.9 ± 0.6	0.9 ± 0.2
Bio5	Maximum temperature in the warmest month (°C)	0	0
Bio6	Minimum temperature in the coldest month (°C)	0	0
Bio7	Temperature annual range (BIO5–BIO6) (°C)	0	0
Bio8	Mean temperature in the wettest quarter (i.e., a period of three months) (°C)	0.1 ± 0.1	0.4 ± 0.1
Bio9	Mean temperature in the driest quarter (°C)	0.1 ± 0.1	0.6 ± 0.3
Bio10	Mean temperature in the warmest quarter (°C)	0.1 ± 0.2	0.4 ± 0.1
Bio11	Mean temperature in the coldest quarter (°C)	0	0
Bio12	Annual precipitation (mm)	26.8 ± 2.6	16.0 ± 1.5
Bio13	Precipitation in the wettest month (mm)	0	0
Bio14	Precipitation in the driest month (mm)	37.4 ± 2.5	4.7 ± 0.5
Bio15	Precipitation seasonality (coefficient of variation)	0.4 ± 0.4	0.3 ± 0.1
Bio16	Precipitation in the wettest quarter (mm)	0.4 ± 0.2	0.2 ± 0.1
Bio17	Precipitation in the driest quarter (mm)	0	0
Bio18	Precipitation in the warmest quarter (mm)	0	0
Bio19	Precipitation in the coldest quarter (mm)	0	0

The data are shown as means ± standard deviations (*n* = 10).

**Table 2:** Habitat suitability for *S. angulatus* in the global terrestrial ecosystem, mainland China, and Liaoning Province under current and future climate change conditions.

	Climate Scenarios	Habitat Suitability			
		<0.1	0.1–0.3	0.3–0.5	>0.5
Global (%)	Contemporary era (1970–2000)	89.5 ± 0.1	6.4 ± 0.1	2.9 ± 0.1	1.2 ± 0.1
	2050s under RCP2.6	84.7 ± 0.1	7.8 ± 0.1	5.1 ± 0.1	2.4 ± 0.1
	2090s under RCP2.6	84.1 ± 0.2	8.1 ± 0.1	5.5 ± 0.1	2.3 ± 0.1
	2050s under RCP8.5	82.3 ± 0.1	8.8 ± 0.1	6.2 ± 0.1	2.8 ± 0.1
	2090s under RCP8.5	78.2 ± 0.1	9.1 ± 0.1	8.8 ± 0.1	3.9 ± 0.1
China (%)	Contemporary era (1970–2000)	66.4 ± 0.5	14.9 ± 0.5	18.1 ± 0.3	0.6 ± 0.2
	2050s under RCP2.6	57.9 ± 0.6	18.5 ± 0.5	22.4 ± 0.6	1.2 ± 0.5
	2090s under RCP2.6	57.0 ± 0.5	19.1 ± 0.5	22.9 ± 0.8	1.1 ± 0.4
	2050s under RCP8.5	54.9 ± 0.4	17.3 ± 0.9	26.3 ± 0.8	1.6 ± 0.8
	2090s under RCP8.5	51.2 ± 0.3	21.5 ± 1.2	25.9 ± 0.9	1.5 ± 0.8
Liaoning (%)	Contemporary era (1970–2000)	3.1 ± 0.9	44.6 ± 1.3	52.2 ± 1.7	0.1 ± 0.1
	2050s under RCP2.6	0	7.0 ± 1.7	65.7 ± 10.5	27.3 ± 10.4
	2090s under RCP2.6	0	4.1 ± 1.1	61.7 ± 9.0	34.2 ± 9.1
	2050s under RCP8.5	0	1.5 ± 1.5	59.8 ± 12.7	38.8 ± 13.3
	2090s under RCP8.5	0	1.6 ± 2.4	71.3 ± 9.4	27.0 ± 10.2

Habitat suitability scores for *S. angulatus* were generated via the *MaxEnt* model and were classified as unsuitable (<0.1), poorly suitable (0.1–0.3), moderately suitable (0.3–0.5), and highly suitable (>0.5). The data are shown as means ± standard deviations (*n* = 10).

**Table 3:** Area changes with habitat suitability greater than 0.1 at global and continental scales under different future climate change scenarios.

Items	Climate Change Scenarios	Area Change						
		World	Asia	Africa	North America	South America	Europe	Oceania
Expansion	2050s under RCP2.6	5680 (32.11%)	1021 (26.01%)	84 (19.34%)	2225 (37.89%)	117 (12.16%)	1941 (32.47%)	293 (56.1%)
	2090s under RCP2.6	6088 (33.65%)	1357 (31.87%)	83 (19.14%)	2045 (35.93%)	118 (12.27%)	2289 (36.19%)	196 (46.11%)
	2050s under RCP8.5	8514 (41.49%)	2117 (42.18%)	54 (13.34%)	2998 (45.11%)	128 (13.19%)	3012 (42.73%)	206 (47.35%)
	2090s under RCP8.5	15,577 (56.47%)	4739 (62.02%)	59 (14.46%)	6160 (62.81%)	297 (26.08%)	4092 (50.34%)	229 (50.07%)
Stable	2050s under RCP2.6	11,101 (62.76%)	2691 (68.59%)	217 (49.94%)	3576 (60.91%)	469 (48.95%)	3951 (66.1%)	197 (37.74%)
	2090s under RCP2.6	10,980 (60.67%)	2668 (62.62%)	178 (40.99%)	3542 (62.22%)	443 (46.1%)	3946 (62.38%)	204 (48.06%)
	2050s under RCP8.5	10,742 (52.35%)	2644 (52.68%)	147 (36.27%)	3485 (52.45%)	380 (39.2%)	3914 (55.53%)	172 (39.67%)
	2090s under RCP8.5	8993 (32.6%)	1983 (25.95%)	66 (15.99%)	2874 (29.31%)	297 (26.09%)	3616 (44.48%)	157 (34.35%)
Reduction	2050s under RCP2.6	906 (5.12%)	212 (5.4%)	134 (30.72%)	70 (1.2%)	373 (38.89%)	85 (1.43%)	32 (6.16%)
	2090s under RCP2.6	1028 (5.68%)	235 (5.51%)	173 (39.87%)	105 (1.85%)	400 (41.63%)	91 (1.44%)	25 (5.83%)
	2050s under RCP8.5	1265 (6.16%)	258 (5.15%)	204 (50.39%)	162 (2.43%)	462 (47.6%)	123 (1.74%)	56 (12.99%)
	2090s under RCP8.5	3014 (10.93%)	919 (12.03%)	285 (69.55%)	773 (7.88%)	545 (47.82%)	421 (5.18%)	71 (15.58%)

Note: Values preceding the brackets denote the number of *S. angulatus* occurrence records. Percentages in brackets denote the proportions of the area changed under climate change scenarios relative to the area under current climate conditions.

## Data Availability

Data are contained within the article and the Supplementary Materials.

## Supplementary Materials

The following supporting information can be downloaded at https://www.mdpi.com/article/10.3390/plants14172745/s1. Figure S1: ROC curves of sensitivity vs. specificity for *S. angulatus*; Figure S2: Locations (red points) of occurrence records of *S. angulatus* in Liaoning Province; Figure S3: Pearson’s correlation coefficients (r) among climatic variables across the 2083 sites.
