# Supplementary material for: Assessment of Habitat Suitability for the Invasive Vine Sicyos angulatus Under Current and Future Climate Change Scenarios
Source: Plants (Basel). 2025 Sep 2;14(17):2745. doi: 10.3390/plants14172745 (PMC12430053; doi:10.3390/plants14172745)

Figure SI

Figure S1

ROC curves of sensitivity vs. specificity for *S. angulatus*. We incorporated 10 replicates in our modeling process to assess variability in the results.

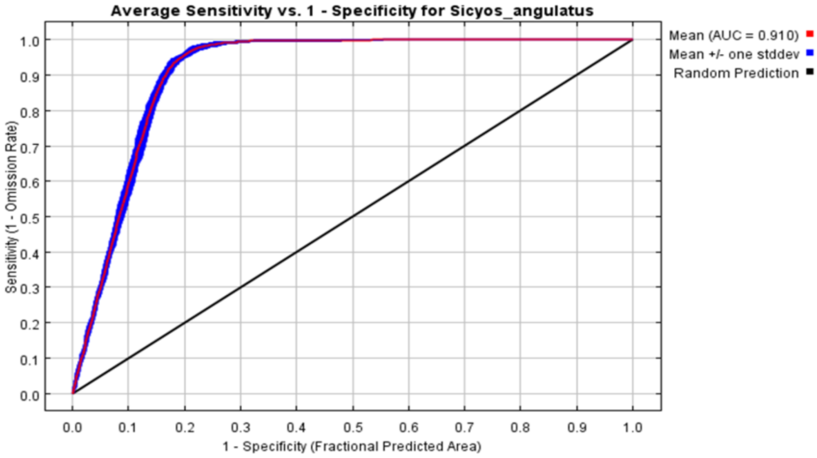

Figure S2

Locations (red points) of occurrence records of *S. angulatus* in Liaoning Province.

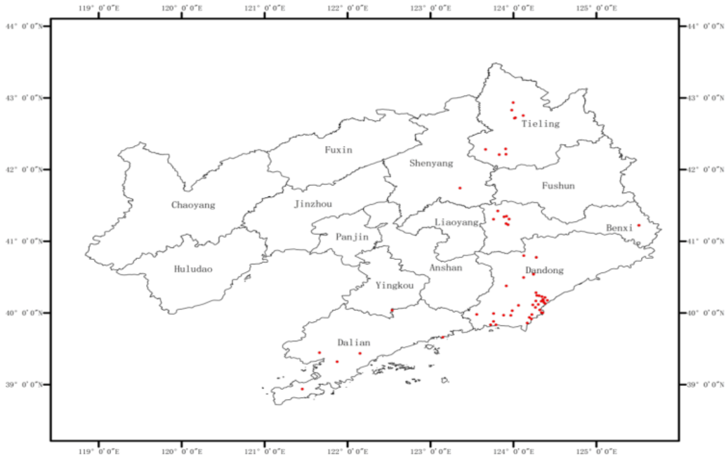

Figure S3

Pearson's correlation coefficients (r) among climatic variables across the 2,083 sites. Bio1, average annual temperature (°C); Bio2, mean diurnal range (mean monthly value of (maximum temperature - minimum temperature)) (°C); Bio3, isothermality (BIO2/BIO7) (×100); Bio4, temperature seasonality (standard deviation ×100); Bio5, maximum temperature in the warmest month (°C); Bio6, minimum temperature in the coldest month (°C); Bio7, temperature annual range (BIO5-BIO6) (°C); Bio8, mean temperature in the wettest quarter (i.e., a period of three months) (°C); Bio9, mean temperature in the driest quarter (°C); Bio10, mean temperature

in the warmest quarter (°C); Bio11, mean temperature in the coldest quarter (°C); Bio12, annual precipitation (mm); Bio13, precipitation in the wettest month (mm); Bio14, precipitation in the driest month (mm); Bio15, precipitation seasonality (coefficient of variation); Bio16, precipitation in the wettest quarter (mm); Bio17, precipitation in the driest quarter (mm); Bio18, precipitation in the warmest quarter (mm); Bio19, precipitation in the coldest quarter (mm). Blue and red denote for positive and negative relationships, respectively.

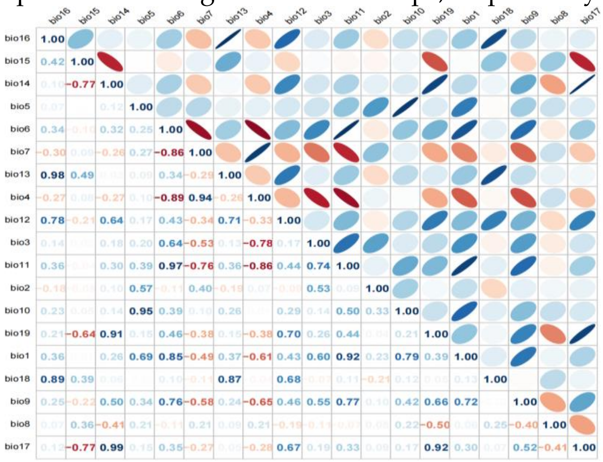

Supplement: Supplementary file 1 [file plants-14-02745-s001.zip › plants-3729534-supplementary.pdf]
